# Acute type A aortic dissection in a patient with COVID-19

**DOI:** 10.2217/fca-2020-0103

**Published:** 2020-09-18

**Authors:** Shiva Tabaghi, Mohammad Ali Akbarzadeh

**Affiliations:** ^1^Cardiovascular Research Center, Shahid Beheshti University of Medical Sciences, Tehran, Iran

**Keywords:** aortic dissection, cardiac surgery, novel coronavirus

## Abstract

The novel coronavirus spread all over the world in 2019 and became a serious international health concern of this century. Coronavirus disease 2019 (COVID-19) had a wide range of clinical manifestations; it can cause mild-to-severe multiorgan diseases, mostly affecting the respiratory system, but cardiovascular symptoms and complications are also frequently presented in COVID-19 patients. Herein, we report a type A aortic dissection in a confirmed case of COVID-19.

The 2019 novel coronavirus (2019-nCoV) has caused an outbreak of respiratory illness since December 2019, beginning in Wuhan, China, and has since spread all over the world as a pandemic. The 2019-nCoV commonly affects upper and lower respiratory airways and presents with a sore throat, sputum production, sneezing, dry coughs, dyspnea, rhinorrhea, fever, pneumonia and SARS coronavirus 2 (SARS-CoV-2) [1–3]; also, the 2019-nCoV can cause myalgia, arthralgia, gastrointestinal presentation (diarrhea, vomiting, loss of appetite, abdominal pain and liver injury with raised enzymes), olfactory and gustatory dysfunctions, CNS involvement and neurological manifestations (headache, dizziness, change in mental status and meningeal signs), metabolic and electrolyte derangements and multiorgan failure [3–5].

However, some patients initially present cardiovascular symptoms such as palpitations and chest tightness. The complications of cardiovascular involvement have also been reported, including acute pericarditis, left ventricular dysfunction (heart failure), cardiac arrest, acute coronary syndromes, myocardial infarction and thromboembolic events including deep vein thrombosis, thrombosis of extracorporeal circuits for continuous veno-venous hemofiltration, central venous catheter-associated thrombosis and pulmonary embolism. Complications and in-hospital death among COVID-19 patients were increased in patients with a history of cardiovascular disease but 11.8% of COVID-19 patients with no history of pre-existing cardiovascular disease had cardiac injury and arrest, resulting in death [6–10]. In this article, we present a patient with COVID-19 that was complicated by aortic dissection.

## Case presentation

A 47-year-old woman was admitted to Ibn Sina Hospital, Noorabad, Lorestan (Iran) on 20 April 2020, with a history of dyspnea, shakes, dry coughs and bloody diarrhea from 2 weeks before admission. The patient had no history of hypertension, surgical procedures, cardiac catheterization, inherited disorders of connective tissue, cocaine usage or other underlying diseases.

**Figure 1.**
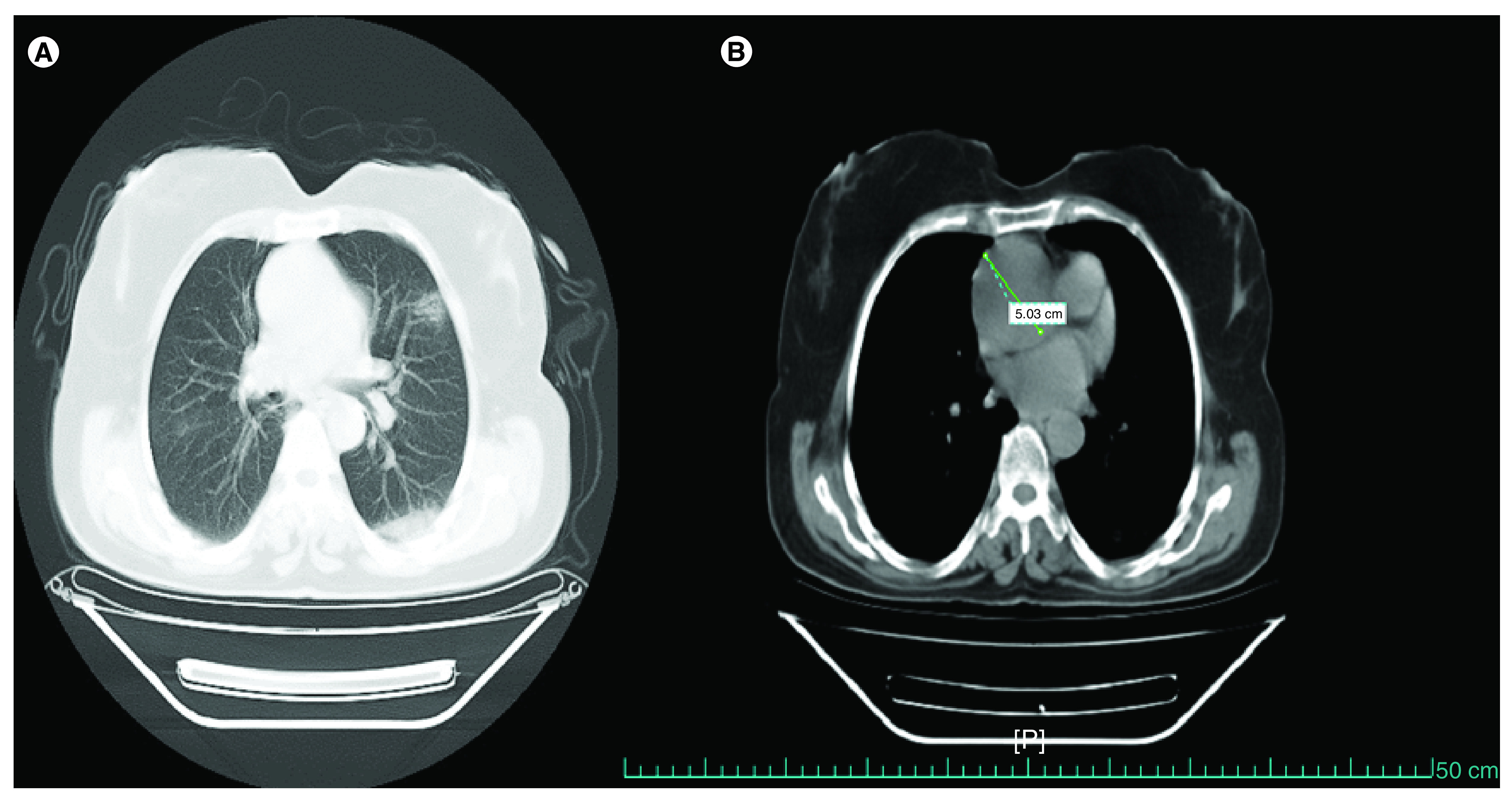
Chest computed tomography. **(A)** Patchy ground glass opacities and consolidation and **(B)** dilated aortic root up to 5 cm.

Her chest CT scan on the first day of admission was carried out without contrast because she had no signs or symptoms of aortic dissection. The CT scan showed patchy ground-glass opacities and consolidation in the sub pleural region of both lungs, more prominently in the superior segment of the left lower lobe and a dilated ascending aorta of about 50 mm (Figure 1). Her blood pressure was 110/75 mmHg in both arms, the heart rate was 80 beats per min, the temperature was 36.1°C, the respiratory rate was 20 breaths per min and the arterial oxygen saturation was 85% without O_2_ supplement and 97% with O_2_ supplement, elevated C-reactive protein was detected. The real-time reverse transcriptase PCR assay was positive for the nasopharyngeal swab of SARS-Cov-2 virus nucleic acid. The ECG showed no abnormalities. She was admitted to the COVID-19 ward and treated with oseltamivir and hydroxychloroquine from the first day of admission. During hospitalization, she had no chest pain, her general conditions, such as respiratory and hemodynamic statuses were stable; no sign of organ failure was detected.

On the 8th day of admissions, at 2 pm, she developed chest pain, loss of consciousness and bradycardia, and was intubated. Her vital signs were blood pressure: 80/pulse mmHg, heart rate: 45 beats per min and the ECG showed junctional rhythm. Before this event as the patient was hemodynamically stable, no vasopressor drugs were prescribed for her.

**Figure 2.**
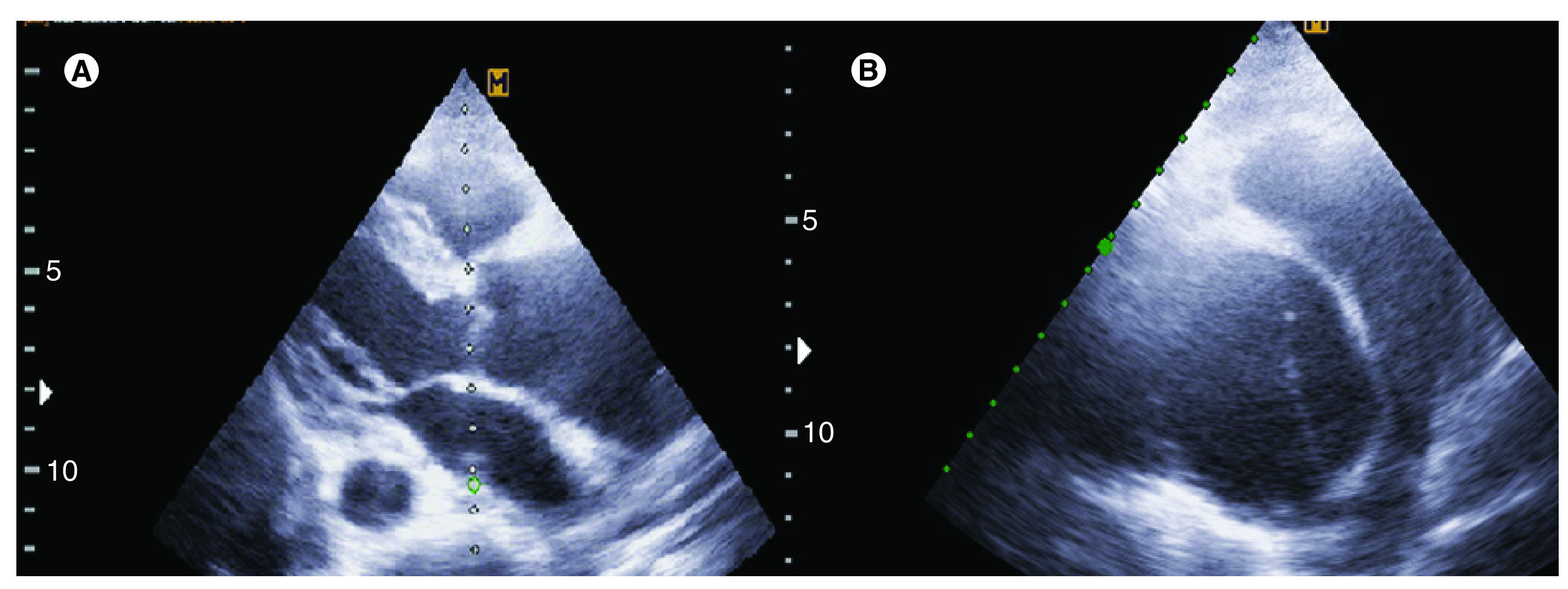
Transthoracic echocardiography. **(A)** Dilated ascending aorta in parasternal long-axis view and **(B)** intimal flap is seen in ascending aorta in short-axis view.

Transthoracic echocardiography revealed normal left and right ventricular size and systolic function, a tricuspid aortic valve with severe aortic insufficiency, dilated aortic root and ascending aorta, a double lumen with an intimal flap of the ascending aorta. No coarctation of the aorta was detected (Figure 2).

The patient was a candidate for emergency cardiac surgery, but due to instability, she expired before being transferred to the operating room.

## Discussion

We reported a case of single strand-positive RNA novel coronavirus pneumonia that presented with bloody diarrhea, dry coughs and dyspnea and developed acute type A aortic dissection. She expired before being transferred for operation. Soon after the first case of COVID-19 was reported in China, this novel virus affected most countries around the world. In 2002 the SARS-CoV and in 2012, the middle east respiratory syndrome coronavirus were presented and had some similarity to this novel virus. Information about clinical presentation and complications of COVID-19 including respiratory, cardiovascular, gastrointestinal and otolaryngological symptoms and neurological involvement had been developing since the initial identification [2–5,11,12].

Many articles have been published to describe cardiovascular involvement such as cardiac insufficiency and myocarditis. Arrhythmias due to the prescription of antiviral drugs and pharmacological agents such as hydroxychloroquine or chloroquine have also been reported in some studies. Patients with COVID-19 and cardiovascular diseases have poor prognosis [10,13,14].

Many cases of elective surgery have been postponed due to the current pandemic, but some elective and especially emergency cardiac surgeries cannot be delayed. Moreover, a heavy burden of mortality of cardiac surgery may be considered in patients with COVID-19 and the unavailability of information and the lack of specific guidelines for elective and emergency surgical interventions make it difficult to manage these patients, so developing a standard and safe protocol is needed [15–17].

There is a concern about the relationship between a viral infection and aortic dissection. Recently an increased admission for aortic dissection and higher in hospital mortality of urgent aortic dissection surgery during the influenza season compared with noninfluenza season were noticed. It has been detected that the number of patients with acute aortic dissection were increased in winter months, regardless of climate. Inflammatory, immune-mediated injury, increased sympathetic activity or medication uses are some of the reasons that may contribute to aortic dissection a patient with influenza infection but after influenza vaccination, the rate of dissection was lowered [18–21].

In previous coronavirus and influenza pandemics, accelerated rate of cardiovascular complication occurred; therefore the association of the new coronavirus with cardiovascular complications such as aortic dissection should be suspected. Although the rapid mortality rate, low incidence and limitation of detecting cases in aortic dissection, may cause difficulty in confirming this association [18–21].

However, our case was a known COVID-19 patient that was hospitalized, and on the 8th day of admission, the case was complicated by aortic dissection. Although her chest CT scan at the time of admission showed ascending aorta dilation, the medical staff focus was on the management of COVID infection. We do not know whether COVID-19 was a trigger for the aortic dissection or if it was a coincidental finding.

Fukuhara *et al.* report a case of acute type A (DeBakey type I) aortic dissection presenting with chest and abdominal pain. On the sixth postoperative day, a positive COVID-19 PCR test was confirmed from the nasopharynx swab. As in our case, this article could not confirm the association between COVID-19 and aortic dissection, but the authors similarly acknowledge the need to develop practical and clinical guidelines for management of urgent/emergent life threatening surgery during the COVID-19 outbreak [22].

At any rate, this case presentation emphasizes the concomitant management of all the problems of COVID-19 patients, and not merely viewing them as purely infectious patients.

## Conclusion

Cardiovascular damage has been reported in COVID-19 patients with potentially severe diseases. It seems that cardiovascular protection should be considered during treatment for COVID-19 patients, and cardiothoracic surgery protocols must be practically defined during the COVID-19 pandemic.

**Summary points**

- We presented a COVID-19 patient that was complicated by aortic dissection. The association between this novel coronavirus and aortic dissection should be considered.
- The first confirmed case of the 2019 novel coronavirus was reported from East of Asia and quickly spread across the entire world, causing a pandemic.
- Our information was limited to describe the clinical presentations of this novel virus but it seems to cause multiorgan disease and organ failures. The human respiratory system is the major target of COVID-19 but other organs such as cardiovascular, gastrointestinal, CNS may be affected.
- COVID-19 caused myocardium and vascular damage and the history of cardiovascular disease in patients with COVID-19, increased the risk of complications and mortality. Therefore cardiovascular protection during COVID-19 treatment should be considered.
- Recently an association between viral syndromes and aortic dissection, especially in winter months were noticed so vaccination may reduce the incidence of dissection.
- In the current pandemic, practical and safe guidelines have been required for management of elective and urgent/emergent cardiac surgeries.
